# Low immediate postoperative serum-cortisol nadir predicts the short-term, but not long-term, remission after pituitary surgery for Cushing’s disease

**DOI:** 10.1186/s12902-015-0055-9

**Published:** 2015-10-25

**Authors:** Jon Ramm-Pettersen, Helene Halvorsen, Johan Arild Evang, Pål Rønning, Per Kristian Hol, Jens Bollerslev, Jon Berg-Johnsen, Eirik Helseth

**Affiliations:** Department of Neurosurgery, Oslo University Hospital, Oslo, Norway; Faculty of Medicine, University of Oslo, Oslo, Norway; Section of Specialized Endocrinology, Medical Clinic B, Oslo University Hospital, Oslo, Norway; The Intervention Centre, Oslo University Hospital, Oslo, Norway

**Keywords:** Pituitary adenoma, Cushing’s disease, Transsphenoidal surgery, Cortisol measurement, Endocrine remission

## Abstract

**Background:**

Cushing’s disease is an ACTH-producing pituitary adenoma, and the primary treatment is microscopic or endoscopic transsphenoidal selective adenectomy. The aims of the present study were to evaluate whether the early postoperative S-cortisol level can serve as a prognostic marker for short- and long-term remission, and retrospectively review our own short and long term results after surgery for Cushing’s disease.

**Methods:**

This single centre, retrospective study consists of 19 consecutive patients with Cushing’s disease who underwent transsphenoidal surgery. S-cortisol was measured every 6 h after the operation without any glucocorticoid replacement. We have follow-up on all patients, with a mean follow-up of 68 months.

**Results:**

At the three-month follow-up, 16 patients (84 %) were in remission; at 12 months, 18 (95 %) were in remission and at the final follow-up (mean 68 months), 13 (68 %) were in remission. Five-years recurrence rate was 26 %. The mean postoperative S-cortisol nadir was significantly lower in the group of patients in remission than in the non-remission group at 3 months, but there was no difference between those in long-term remission compared to those in long-term non-remission. The optimal cut-off value for classifying 3-month remission was 74 nmol/l.

**Conclusion:**

We achieved a 95 % 1-year remission rate with transsphenoidal surgery for Cushing’s disease in this series of consecutive patients. However, the 5-year recurrence rate was 26 %, showing the need for regular clinical and biochemical controls in this patient group. The mean postoperative serum-cortisol nadir was significantly lower in patients in remission at 3 months compared to patients not in remission at 3 months, but a low postoperative S-cortisol did not predict long-term remission.

## Background

Cushing’s disease is caused by a pituitary adenoma that hyper secretes adrenocorticotropic hormone (ACTH), and it was first described by Harvey Cushing in 1932 [1]. Untreated Cushing’s disease is associated with significant morbidity and mortality [2]. The primary treatment is transsphenoidal selective adenectomy, either microscopic or endoscopic. The remission rate after selective microadenectomy varies greatly between 55 and 95 % [3–8], and the recurrence rate after initial remission of Cushing’s disease is reported to be in the range 5–38 % 10 years after surgery [9, 10]. A second transsphenoidal surgery is an option for patients who are not initially cured or with recurrent disease, but it is associated with a lower success rate than primary surgery and more surgery-related complications [11, 12].

There is no international consensus on the definition of remission for Cushing’s disease after transsphenoidal surgery. The large variations in the remission rates after surgery might reflect this lack of consensus. The most commonly used remission criteria are the following: clinical resolution of symptoms, normal low dose dexamethasone suppression test (DST) (2 mg/d for 48 h), normal urinary free cortisol level and normal late night salivary cortisol [13].

Recent publications on Cushing’s disease have suggested that early postoperative serum-cortisol (S-cortisol) levels can predict remission [14–16]. The main aim of the present study was to evaluate the role of the early postoperative S-cortisol level as a prognostic marker for short- and long-term remission.

## Methods

This is a single centre, retrospective study of 19 consecutive patients with Cushing’s disease who underwent operation performed by a single neurosurgeon (JRP) at Oslo University Hospital (OUS), Oslo, Norway between 2003 and 2008.

### Preoperative diagnostic work-up

The initial endocrine evaluation was performed at a specialised endocrinology centre at OUS. Patients with clinical features and a baseline laboratory test indicative of Cushing’s syndrome were evaluated with 24-h urinary free cortisol, late night salivary cortisol, 48-h, low dose dexamethasone suppression test (DST) (2 mg/d for 48 h) and corticotrophin-releasing hormone (CRH) test. Once the diagnosis of ACTH-dependent Cushing’s syndrome was established [17], a multidisciplinary team, consisting of an endocrinologist, radiologist, oncologist and neurosurgeon, discussed the patients and established the indication for surgery. All patients underwent 1.5 tesla MRI scans after a dedicated pituitary protocol, and those with initial normal scans were investigated with dynamic sequences. When indicated, inferior petrosal sinus sampling (IPSS) was performed to establish the diagnosis of Cushing’s disease. When IPSS was performed ACTH was measured both before and after intravenous CRH administration. A central-to-peripheral ratio of more than two before CRH administration, or more than three after, was interpreted as positive for Cushing’s disease [18].

### Surgical technique

All patients underwent operation while under general anaesthesia, with total intravenous anaesthesia in most cases. The standard microsurgical endonasal transseptal approach was used prior to 2006. Through the right nostril, an incision was made in the anterior part of the septum, and the mucosa was dissected from the septal cartilage and bone. The anterior wall of the sphenoid sinus was opened bilaterally and any septum in the sphenoid sinus was removed as necessary. The floor of the sella was opened with a high-speed drill. The tumour was removed with standard instruments, such as curettes, suction and micro forceps. After tumour removal, the floor of the sella was reconstructed with two layers of Neuropatch® (BBraun, Tutlingen, Germany) and the septal bone to reconstruct the floor of the sella, which was sealed with fibrin glue and gelfoam. In a few patients with small nostrils (paediatric cases), a sublabial approach was performed. After 2006, the endoscopic endonasal transsphenoidal approach became standard procedure. One or both nostrils were entered according to the available space and need for exposure during the surgery. We used standard Storz endoscopes (Karl Storz GmbH & Co, Tuttlingen, Germany) (180/4 mm), with 0^0^, 30^0^ and 45^0^ angulations coupled to cameras, in the later period of the study to HD cameras. In the first cases, we used a fixed endoscope support, whereas in the later cases, the endoscope was handled freehand to increase flexibility during surgery. Image-guided navigation (BrainLab AG, Feldkirchen, Germany) was used in some procedures. Tumour resection was performed with standard surgical instruments, such as dissectors, curettes, suction and micro forceps, dependent on the tumour size and firmness. In tumours with parasellar extension, we routinely used an ultrasonic Doppler probe (Mitzhuo Medical Inc, Tokyo, Japan) to localise the internal carotid artery. The surgical corridor was closed with two layers of Duragen (Integra LifeScience Corporation, New Jersey, USA) and a Porex plate (Porex Corporation, Atlanta, Georgia, USA) for rigid reconstruction of the sellar floor. One dose of cefalotin was administered intravenously before the operation. No glucocorticoids were given pre- or perioperatively.

### Surgery-related complications

The following complications were recorded: mortality, CSF-leakage, vascular injury, infection, new neurological deficits and new hormone deficiencies, including ADH deficiency.

### Endocrine evaluation immediately after surgery and before discharge

No exogenous glucocorticoids were given postoperatively, and the serum levels of cortisol were measured every 6 h at fixed hours (12, 18, 24, 06, 12, and so forth), starting immediately after the end of surgery. If the patient showed clinical symptoms or signs of hypocortisolaemia (headache, nausea, fatigue, low serum sodium, fever, and hypotension) or had S-cortisol levels below 100 nmol/l, exogenous glucocorticoids were administered, initially per oral cortisone acetate 100 mg × 2, and no further measurements of S-cortisol was done. Patients without clinical symptoms of hypocortisolaemia who did not reach a nadir of 100 nmol/dl were prescribed exogenous glucocorticoids at discharge from the hospital for use as required. The S-cortisol levels were measured for 1 to 4 days after surgery. We used a S-cortisol nadir of <50 nmol/l, <100 nmol/l and <200 nmol/l to predict remission at the different endpoints, and we calculated the sensitivity and specificity at each time point.

### Definitions of remission and recurrence

Remission was defined as relief from clinical symptoms, urinary free cortisol below threshold, late night salivary cortisol below threshold, and suppression to <50 nmol/l of S-cortisol on 1 mg overnight or 2 mg/D, 48 h DST. The definition of recurrence is difficult, and it must be stressed that this is an integrated clinical and biochemical assessment. Relapse of clinical symptoms, such as cutaneous symptoms (red striae, easy bruising and thin skin), central obesity and proximal muscle weakness, are strong indicators of disease recurrence, but these symptoms often appear late and long after biochemical tests indicate relapse from the disease. We used elevated morning S-cortisol, elevated late night salivary cortisol, elevated 24 h urinary free cortisol, elevated morning serum cortisol and inadequate suppression of cortisol after DST as the criteria for recurrence.

### Follow-up

Patients were seen in the outpatient ward of the section of specialised endocrinology at 6 weeks, 3 months and 6 months after surgery and thereafter at least once a year.

### Statistical analysis

The data are summarised with counts, percentages, means and medians as appropriate. T-tests robust for unequal variances are used. Due to the correlational structure of the repeated intra-individual cortisol measurements, the confidence intervals of Figs. 2 and 3 were calculated according to Morey [19]. The time trend of cortisol was investigated using logistic regression and the area under the curve (AUC) of the cortisol values over time, nadir values and slope until nadir. The nadir value was further investigated as a classifier for remission using receiver-operating-characteristics (ROC) curves. The optimal cut-off was calculated by finding the maximum Youden index over all nadir values. The confidence interval for the AUC in the ROC was calculated using bootstrapping techniques. A confidence interval for the ROC AUC excluding 0.5 was considered significant.

Excel 2010 (Microsoft) and R version 3.0.3 [20] were used for all statistical analysis. P-values <0.05 were considered significant.

## Results

Nineteen consecutive patients with clinically and biochemically verified Cushing’s disease were included in this study. There were 13 females (68 %). The median age was 38.0 years (range 13–66 years). Three patients had undergone a previous operation and had biochemical and neuroradiological residual tumour. They were primarily operated in 1995, 1999 and 2000. All patients are represented only once in this material.

Preoperative MRI showed a microadenoma in 13 patients (68 %), macroadenoma in two patients (11 %) and no tumour in four patients (21 %). In cases without visible tumour, the diagnosis of Cushing’s disease was based on demonstrating ACTH-dependent Cushing’s syndrome in combination with positive IPSS. Details on the pre and intraoperative findings are given in Table 1.

**Table 1:** Pre and intraoperative findings and postoperative result

Pt	MRI	Histology	Result			Cortisol substitution		
			3 months	12 months	Last follow-up	3 months	12 months	Last follow-up
1	Neg	Adenoma	Remission	Remission	Remission	Yes	No	No
2	Micro^a^	Adenoma	Remission	Remission	Remission	Yes	Yes	No
3	Macro^b^	Adenoma	Remission	Remission	Remission	No	No	No
4	Micro	No tumour	Persistent	Remission	Remission	No	No	No
5	Neg	No tumour	Remission	Remission	Remission	Yes	Yes	No
6	Micro	No tumour	Remission	Remission	Recurrence	No	No	No
7	Macro	Adenoma	Remission	Remission	Recurrence	Yes	No	No
8	Micro	Adenoma	Remission	Remission	Remission	Yes	Yes	No
9	Micro	Adenoma	Remission	Remission	Remission	Yes	Yes	Yes
10	Micro	Adenoma	Remission	Remission	Remission	Yes	Yes	No
11	Micro	Adenoma	Remission	Remission	Remission	Yes	No	No
12	Micro	No tumour	Remission	Remission	Remission	Yes	Yes	Yes
13	Micro	Adenoma	Remission	Remission	Remission	Yes	No	No
14	Neg	No tumour	Remission	Remission	Recurrence	No	No	No
15	Micro	Adenoma	Remission	Remission	Recurrence	No	No	No
16	Micro	No tumour	Remission	Remission	Recurrence	Yes	No	No
17	Micro	No tumour	Persistent	Remission	Remission	No	No	No
18	Neg	No tumour	Persistent	Persistent	Persistent	No	No	No
19	Micro	Adenoma	Remission	Remission	Remission	Yes	Yes	Yes

^a^micro < 10 mm

^b^macro ≥ 10 mm

All patients underwent transsphenoidal surgery. Neuropathological examination verified an ACTH-producing adenoma in 11 of the 19 patients (58 %). In the remaining eight patients, either an insufficient amount of material was obtained for reaching a confident conclusion or the pathologist did not identify the sample as tumour.

**Fig. 1:**
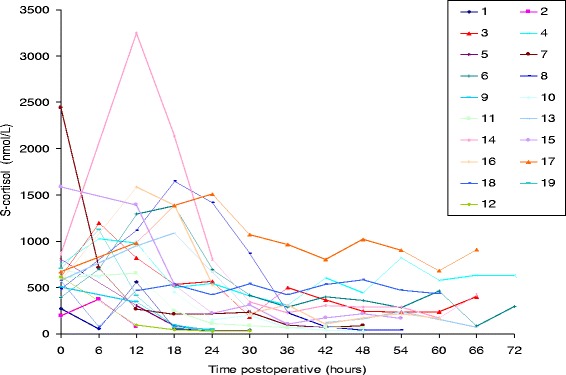
Individual curves of the serum cortisol levels at fixed, 6-h intervals after transsphenoidal surgery for Mb. Cushing

**Fig. 2:**
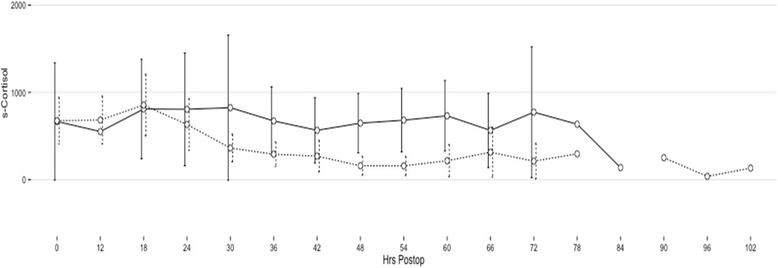
Mean postoperative S-cortisol values at given hours after surgery. The dense line represents patients who are not in remission at the 3-month follow-up, and the dotted line represents patients in remission at the 3-month follow-up. *P* < 0.05 at 54 and 60 h after surgery. Units on the y-axis is nmol/l

**Fig. 3:**
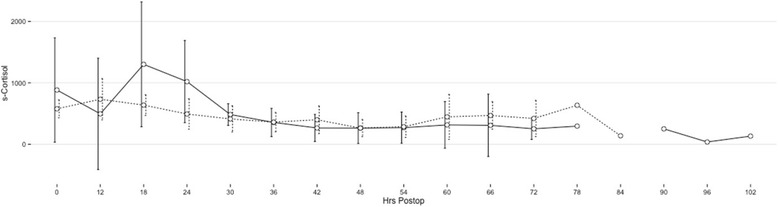
Mean postoperative S-cortisol values at given hours after surgery. The dense line represents patients who were not in remission at the last follow-up (68 months) and dotted line represents patients in remission at the last follow-up. Units on the y-axis is nmol/l

At the 3-month follow-up, 84 % were in remission; at the 12-month follow-up, 95 % were in remission and at the final follow-up (68 months), 68 % were in remission. A summary of the endocrinological outcome at 3 and 12 months as well as in the long-term is given in Table 2. The format of this table is the same as in the publication by Starke et al., facilitating meta-analyses [9]. The individual S-cortisol curves of all patients after surgery are plotted in Fig. 1. There is considerable inter-individual variation, but in the first three postoperative days, there was a tendency towards lower values as the time from surgery increased. In Fig. 2, the postoperative S-cortisol values were stratified according to remission at 3 months. Using paired T-tests, there are significant differences between the curves at 54 and 60 h after surgery, but statistical curve analysis did not reveal any significant difference between the curves. In Fig. 3, the postoperative S-cortisol values are stratified according to remission at the last follow-up. The postoperative S-cortisol values do not predict long-term remission at any time-point. In Table 3, the pre- and intra-operative variables are correlated with short- and long-term remission. The mean S-cortisol nadir is significantly lower in the group of patients in remission at 3 months compared to the group of patients not in remission at 3 months. No such correlation was seen for long-term remission.

**Table 2:** Summarised outcome data

Outcome	Follow-up	Follow-up	Last follow-up
	3-month	12-month	(mean 68 months)
	*n* = 19, *n* (% of *n*)	*n* = 19, *n* (% of *n*)	*n* = 19, *n* (% of *n*)
Overall remission	16 (84)	18 (95)	13 (68)
Hypocortisolaemia	12 (63)	7 (37)	3 (16)
Eucortisolaemia	4 (21)	11 (58)	10 (52)
Hypercortisolaemia	3 (16)	1 (5)	6 (32)

**Table 3:** Pre- and intra-operative findings in relation to the short- and long-term outcome

	3 months		Long term	
	In remission	Non remission	In remission	Non remission
	*n* (%)	*n* (%)	*n* (%)	*n* (%)
No. of patients	16 (84)	3 (16)	13 (68)	6 (32)
Macroadenoma	2 (100)	0	1 (50)	1 (50)
Microadenoma	11 (85)	2 (15)	10 (77)	3 (23)
Negative MRI	3 (75)	1 (25)	2 (50)	2 (50)
Histology neg	5 (63)	3 (38)	4 (50)	4 (50)
Mean postoperative S-cortisol nadir	73.44	298.33^*^	110.15	97.17

^*^ *P* < 0.05

**Fig. 4:**
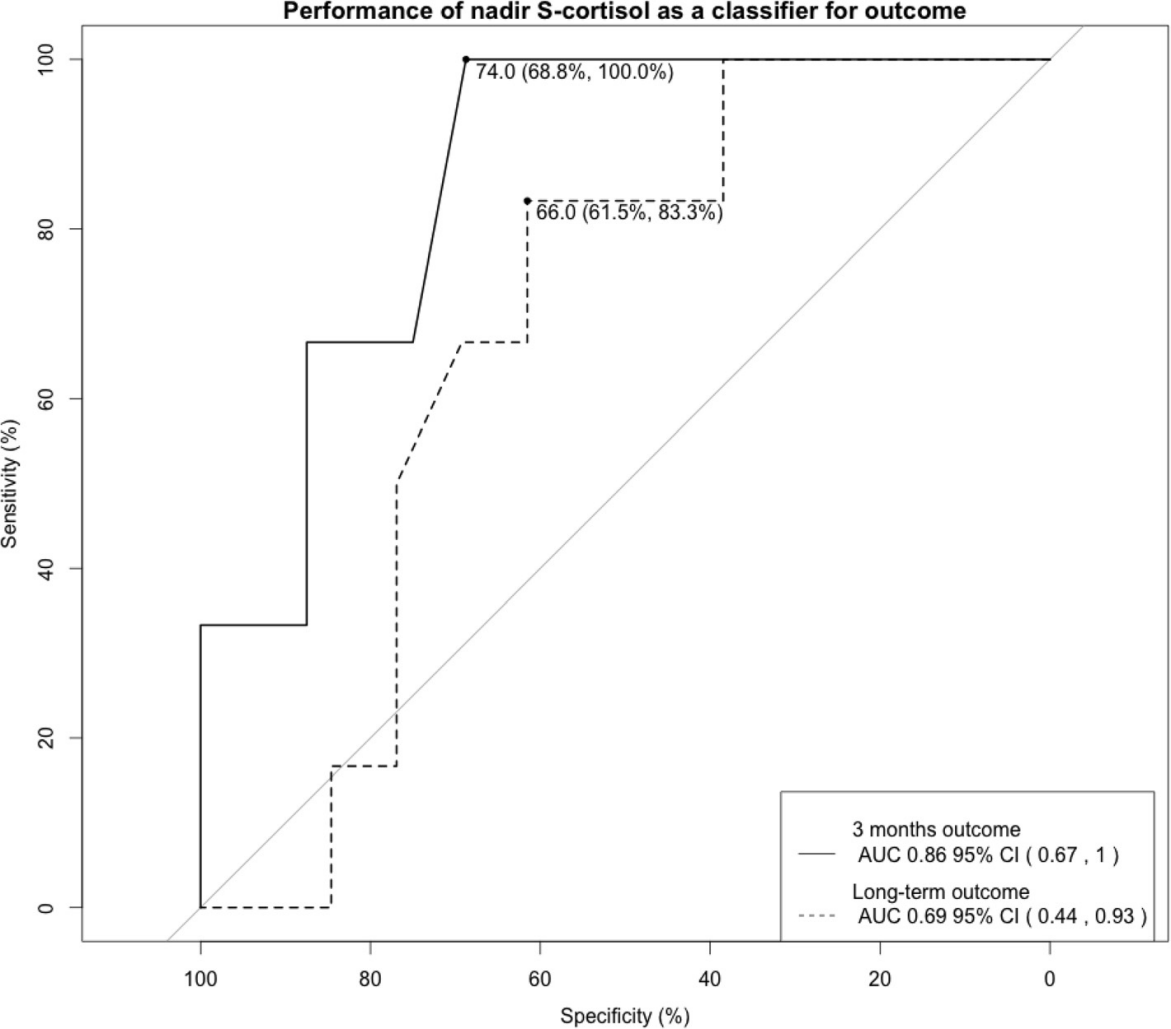
ROC curve demonstrating the performance for the nadir of postoperative S-cortisol in classifying both 3-month and long-term remission. The areas under the curve for the ROC curves are 0.86 (95 % CI (0.67, 1)) and 0.69 (95 % CI (0.44, 0.93)), respectively

In Fig. 4, a ROC curve is displayed, demonstrating the performance for the nadir of postoperative S-cortisol in classifying both the 3-months and long-term remission. The areas under the curve for the ROC curves are 0.86 (95 % CI (0.67, 1)) and 0.69 (95 % CI (0.44, 0.93), respectively. Therefore, the performance of the postoperative S-cortisol nadir in classifying the 3-month and long-term remission was significant and non-significant, respectively. The optimal cut-off value for classifying 3-month remission is 74 nmol/l.

A positive histology, positive preoperative MRI or size of tumour showed no correlation with remission at 3 months or in the long-term.

### Complications

Three patients were substituted for new postoperative hormonal deficiencies; two patients had central hypothyroidism (patient 1 and 19) and one had gonadotrophic deficiency (patient 13). None of the following were present: cerebrospinal fluid leak, vascular injury, infection, new neurological deficits and surgical mortality.

## Discussion

The overall remission rate in this study of 19 patients with clinically and biochemically verified Cushing’s disease was 84 % at 3 months, 95 % at 12 months and 68 % at 68 months follow-up, which is in agreement with other recent published series [9, 21]. A low immediate postoperative S-cortisol value was a predictor for short-term remission but not for long-term remission, which is in contrast to other published studies [9, 14, 16], the reason for this might be that we have long-term follow-up on all patients and a relatively high rate of late recurrences.

### Surgical outcome

The lack of international consensus on the definition of cure after transphenoidal surgery for Cushing’s disease makes the interpretation of reported outcome figures difficult. Most authors use the clinical resolution of symptoms and one or more of the biochemical parameters of normalisation of 24 h urinary free cortisol levels, low late-night saliva cortisol, normal midnight serum cortisol or suppression of cortisol after low-dose dexamethasone test. Due to this lack of consensus and the heterogeneity of patient populations, there is considerable variation in the reported immediate postoperative remission rate, ranging from 55 to 95 % [3–8, 22] The best results are obtained in patients with non-invasive microadenomas with cure rates as high as 93 %, and with a marked reduction in the remission rate with increasing tumour diameter and extrasellar extension [23]. Our results of 84 % in remission at 3 months and 95 % in remission at 1 year are in accordance with the literature. It is worth noting that all of our patients completed follow-up, while in other published series, up to 30 % of the patients were lost to follow-up, which may have biased the results [9, 10].

In this study, with a mean follow-up of 5.7 years (68 months), five out of 19 patients had a late recurrence. This high recurrence rate (26 %) may represent the “natural, but underreported, history of the disease” or the consequence of our surgical practise. Few complications were recorded in this series, with zero surgical complications and three patients with new hormonal deficits, which may reflect a cautious attitude during resection or extirpation of the tumour. On the other hand, long-term follow-up was performed on all patients and will tend to give a higher recurrence rate. In a large study on late recurrences of Cushing’s disease after initial successful surgery, 215 patients were followed for a mean of 45 months, and the actuarial recurrence rate after 5 years was 25.5 % [10]. Atkinson et al. reported a 9.6-year recurrence rate of 22 % [24]. In a newly published report, the recurrence rate was 65.6 % with follow-up of 14 ± 10 years, whereas mean time to recurrence was 2.4 ± 1.7 years [25].

The reported long-term recurrence rates for other endocrine active pituitary adenomas, like growth hormone secreting tumours, are considerably lower, ranging from 2 to 6 % [26, 27]. The higher recurrence rate seen in Cushing’s disease indicates a different tumour biology, which makes surgical cure more difficult.

### Theories for hypo-and hyper-cortisolism after surgery

Different theories try to explain why hypocortisolism is seen after surgery for Cushing’s disease. Hypercortisolism, as part of the disease, suppresses the normal corticotrophes of the pituitary gland, and when the ACTH-producing tumour is removed, the ACTH level and, therefore, cortisol decreases to subnormal levels. The resolution of the suppression of the normal corticotrophes may take as long as 10 to 18 months [21]. If an ACTH-producing tumour is partially resected, the overproduction of ACTH results in reduced cortisol levels that are normalised or even subnormal if the normal corticotroph cells are supressed. This will be interpreted as the patient being cured by surgery, before the tumour regrows and leads to disease recurrence. On the other hand, one may argue that the long standing elevated ACTH levels and, therefore, severely increased cortisol production from the adrenals may result in adrenal hyperplasia that maintains lasting cortisol hypersecretion even after successful removal of the ACTH-producing tumour in the pituitary gland [28].

### Can cortisol reaching a nadir during the first 3 days after surgery predict long-term remission?

We wanted to study whether it is possible to establish distinct criteria for predicting long-term remission early, within hours to days, after transsphenoidal surgery. In our series, there was a significant difference in the immediate postoperative S-cortisol nadir between patients in remission and non-remission at 3 months, which was not the case at 12 months or at the last follow-up. Two patients who were not in remission at the three-month follow-up may explain this; they had late remission that continued throughout the whole follow-up (patients 4 and 17). On the other hand, five of the patients who were in early remission had late recurrence (patients 6, 7, 14, 15 and 16). If we exclude the one patient who never went into remission and look at those five patients who where in remission at 3 and 12 months, but had late recurrence of the disease, nothing differed between them and those who remained in long-term remission. The method used for evaluating early postoperative corticotroph function, including serial measurements of S-cortisol without glucocorticoid substitution, is similar to the method described in three previously published studies [14–16]. These studies found that subnormal postoperative levels of S-cortisol were predictive of sustained remission, but follow-up was a relatively short (27 months and 33 months) compared to our study (68 months). We also found that subnormal values of S-cortisol predicted short-term remission, but subnormal levels did not predict long-term remission.

We have calculated the performance of the nadir values of postoperative S-cortisol in classifying remission. We found that the nadir value could be used to discriminate between remission and non-remission at 3 months with an optimal cut-off at 74 nmol/l. The ROC curve for long-term remission indicated insignificant performance. This is in contrast with some other publications that reported cortisol nadirs as high as 160 nmol/l may be predictive of cure [9], while others failed to find a specific cut-off level [14, 16]. Even with a very low cut-off value for the postoperative S-cortisol nadir, such as 100 nmol/l, that has a high specificity; there would still have been late recurrence in our study with 2 out of 19 patients (11 %) after a mean follow-up of 68 months. Esposito reported a 3 % recurrence rate with a cut-off at 140 nmol/l in their study and mean 33 months of follow-up [16]. In our study, two patients achieved long-term remission in spite of their high postoperative S-cortisol values and even though they where not in remission at the 3-month follow-up. With these cut-off values, they would not have been identified as in remission.

Cortisol has a crucial role in maintaining normal physiological homeostasis, especially in relation to stress, and hypocortisolism after surgery may implicate serious complications, such as Addison’s crisis. Therefore, some institutions administer glucocorticoids routinely to all patients undergoing transsphenoidal surgery. For patients undergoing surgery for Cushing’s disease, we have chosen to withhold glucocorticoids and followed the clinical condition closely, while measuring S-cortisol every sixth hour to detect whether or not the patients are in remission. It is pertinent to ask whether the benefit of avoiding glucocorticoids in the few patients who do not need it is worth the risk of the serious hypocortisolaemic symptoms. No serious complications have been reported from well-recognised pituitary centres who use the same protocol as ours [9, 14–16]. A low postoperative S-cortisol value is certainly gratifying for the surgeon, suggesting that adequate surgery has been performed. If glucocorticoids had been given to all our patients, we would have given steroids to 7 patients who were not in need of the medication (4 eucortisolaemic and 3 hypercortisolaemic). With the relatively high rate of remission reported in this study, we question whether it is correct to withhold glucocorticoids after surgery for Cushing’s disease to detect the few patients who are not in remission.

ACTH-producing adenomas belong to what is probably the most challenging subgroup of pituitary adenomas, and new techniques like intraoperative MRI and endonasal endoscopy may help us strive to achieve a complete resection and improve the outcome after surgery. The use of the histological pseudo-capsule as a surgical margin is a promising new technique for achieving radical surgery in pituitary adenomas, originally presented by Oldfield and Vortmeyer [29]. It has since been shown that pseudo-capsule resection produces a faster postoperative decline in cortisol than piecemeal removal, but it does not have as rapid a decline as seen after hypophysectomy [30]. This elegant surgical technique can unfortunately only be studied in a minority of patients, but it will be interesting to see whether the cure rates can be improved and the long-term recurrence rate reduced in surgery for Cushing’s disease.

## Conclusion

We achieved a high remission rate with transsphenoidal surgery for Cushing’s disease in this series of consecutive patients, and 95 % were in remission at one year. The recurrence rate was relatively high with a 5-year recurrence rate of 26 %, demonstrating the need for thorough clinical and biochemical follow-up. At 3 months, the mean S-cortisol nadir was significantly lower in the remission group than in the non-remission group, but low postoperative S-cortisol did not predict long-term cure.

### Ethics

The data protection official at Oslo University Hospital approved this study. Approval number is 2014/17755. As this study is a retrospective quality control study based on anonymous data and as the patients were not subjected to any new tests or visits, it is according to hospital policy not necessary with written informed consent.
